# Foliar nutrient resorption patterns of four functional plants along a precipitation gradient on the Tibetan Changtang Plateau

**DOI:** 10.1002/ece3.3283

**Published:** 2017-08-02

**Authors:** Guangshuai Zhao, Peili Shi, Jianshuang Wu, Dingpeng Xiong, Ning Zong, Xianzhou Zhang

**Affiliations:** ^1^ Key Laboratory of Ecosystem Network Observation and Modelling Institute of Geographic Sciences and Natural Resources Research Chinese Academy of Sciences Beijing China; ^2^ China National Forestry Economics and Development Research Center Beijing China; ^3^ College of Resource and Environment University of Chinese Academy of Sciences Beijing China; ^4^ Functional Biodiversity Dahlem Center of Plant Science Free University of Berlin Berlin Germany

**Keywords:** environmental controls, leaf nutrient resorption, N:P, nitrogen and phosphorus, plant functional group, precipitation gradient, soil nutrient availability, stoichiometry, Tibetan Changtang Plateau

## Abstract

Nutrient resorption from senesced leaves as a nutrient conservation strategy is important for plants to adapt to nutrient deficiency, particularly in alpine and arid environment. However, the leaf nutrient resorption patterns of different functional plants across environmental gradient remain unclear. In this study, we conducted a transect survey of 12 communities to address foliar nitrogen (N) and phosphorus (P) resorption strategies of four functional groups along an eastward increasing precipitation gradient in northern Tibetan Changtang Plateau. Soil nutrient availability, leaf nutrient concentration, and N:P ratio in green leaves ([N:P]_g_) were linearly correlated with precipitation. Nitrogen resorption efficiency decreased, whereas phosphorus resorption efficiency except for sedge increased with increasing precipitation, indicating a greater nutrient conservation in nutrient‐poor environment. The surveyed alpine plants except for legume had obviously higher N and P resorption efficiencies than the world mean levels. Legumes had higher N concentrations in green and senesced leaves, but lowest resorption efficiency than nonlegumes. Sedge species had much lower P concentration in senesced leaves but highest P resorption efficiency, suggesting highly competitive P conservation. Leaf nutrient resorption efficiencies of N and P were largely controlled by soil and plant nutrient, and indirectly regulated by precipitation. Nutrient resorption efficiencies were more determined by soil nutrient availability, while resorption proficiencies were more controlled by leaf nutrient and N:P of green leaves. Overall, our results suggest strong internal nutrient cycling through foliar nutrient resorption in the alpine nutrient‐poor ecosystems on the Plateau. The patterns of soil nutrient availability and resorption also imply a transit from more N limitation in the west to a more P limitation in the east Changtang. Our findings offer insights into understanding nutrient conservation strategy in the precipitation and its derived soil nutrient availability gradient.

## 1 INTRODUCTION

The limitation of key nutrients, nitrogen (N), or phosphorus (P) on plant growth and primary productivity is remarkable especially in alpine and arid biomes (Aerts & Chapin, 2000). In response to limiting resources, plants improve nutrient acquisition from root systems, and/or nutrient resorption from senesced leaves. Nutrient resorption, that is, internal nutrient recycling is one of the most important nutrient conservation mechanisms to increase plant fitness and improve nutrient cycling especially in nutrient‐poor environment (Aerts, 1996). The spatial patterns of leaf nutrient resorption along environmental gradients are long‐term concerns, which shed light on nutrient conservation strategies of plants. Thus, an in‐depth addressing the influence of biophysical factors on nutrient resorption would offer insights into understanding how nutrient conservation responds to environment change.

Nutrient resorption efficiency and proficiency are two important indices of internal nutrient recycling (Aerts, 1996; Killingbeck, 1996). Globally, mean N and P resorption efficiencies from senesced leaves are estimated to be ca. 62% and 65%, respectively (Vergutz, Manzoni, Porporato, Novais, & Jackson, 2012). Nutrient resorption efficiencies are generally higher in nutrient‐limited than in nutrient‐rich environment (Killingbeck, 1996; Yuan & Chen, 2009). And this pattern varies with climate, soil/leaf nutrient and stoichiometry, and plant functional groups (Brant & Chen, 2015). Most studies to date have quantified nutrient resorption in response to one of these factors in local scale. Yet knowledge gap on the roles and contribution of multi‐factors in influencing nutrient resorption still exists along environmental gradients.

Soil nutrient availability (Aerts & Chapin, 2000; Brant & Chen, 2015; Yuan & Chen, 2009) or the nutrients in plant tissues (Kobe, Lepczyk, & Iyer, 2005; Ratnam, Sankaran, Hanan, Grant, & Zambatis, 2008; Vergutz et al., 2012) largely control nutrient resorption efficiency. So plants in nutrient‐limited environment are likely to evolve with specific conservative strategies of the adaptation to low nutrient stress. Soil nutrient availability and leaf nutrient concentration are necessary for estimating nutrient resorption (Aerts & Chapin, 2000; Oleksyn, Reich, Zytkowiak, Karolewski, & Tjoelker, 2003). Moreover, the relative availability or limitation of different elements also affects nutrient resorption (Gusewell, 2004, 2005; Ratnam et al., 2008). For example, the N:P ratio of green leaves ([N:P]_g_) is widely used to describe the relative limitation of N and P on plant growth. Concretely, a higher [N:P]_g_ means that P limitation is comparably higher than N limitation (Gusewell, 2004; Ratnam et al., 2008; Tessier & Raynal, 2003). As a result, P resorption efficiency increases while N resorption efficiency decreases with increasing [N:P]_g_. However, the relative contribution of soil nutrients and leaf stoichiometry to leaf nutrient resorption remains unclear.

In regional and local scale, precipitation is a key factor to affect soil moisture and fertility, thus regulating soil nutriment availability and nutrient resorption (Brant & Chen, 2015). Soil moisture plays an important role in driving biogeochemical cycles in arid or semiarid ecosystems (Austin et al., 2004; Schwinning & Sala, 2004). Not only soil nutrient availability (Drury, Zhang, & Kay, 2003; Paul et al., 2003), but also plant nutrient concentrations (Reich, 2003; Wright & Westoby, 2003) are influenced by precipitation. Thus, nutrient resorption efficiencies exhibit descending trends with increasing precipitation (Meier & Leuschner, 2014; Reed, Townsend, Davidson, & Cleveland, 2012). In the more arid areas, the stronger nitrogen limit and weaker phosphorus limit (Delgado‐Baquerizo et al., 2013; Wardle, 2013) will affect plant growth and internal nutrient cycling. Deciduous shrubs can adapt drought by ways of multiple leaf production cycle in one growing season (Killingbeck, 1992). In addition, N‐fixing legumes are not necessary to increase nutrient resorption to adapt low nutrient caused by drought compared with nonlegumes (Stewart, Kennedy, Landes, & Dawson, 2008). Despite increasing reports on the influence of plant functional traits on nutrient resorption and cycling, there are relatively few studies on the effects of plant functional types on nutrient resorption. Adding water can differently alter the concentrations of N and P in green leaves in experimental vegetation (Lü & Han, 2010), and thus different patterns of N resorption efficiency (NRE) and P resorption efficiency (PRE) along soil water gradient (Lü & Han, 2010; Yuan & Chen, 2009). Therefore, the changing patterns of leaf N and P resorption are not equivalent in different ecosystems. However, some studies reported that soil nutrients availability remains unchanged or even decreases with increasing precipitation (Austin & Sala, 2002; Barrett, McCulley, Lane, Burke, & Lauenroth, 2002). Accordingly, foliar nutrient concentrations are negatively correlated with mean annual precipitation (Han, Fang, Reich, Ian Woodward, & Wang, 2011; Zhan, 2013). Nevertheless, it is still inconclusive in the question whether nutrient resorption is directly dependent on precipitation or indirectly influenced by precipitation through soil nutrient availability. The difference of plant functional type in its plasticity of drought adaptation and nutrient conservation is waiting for more exploration.

Overall, most studies so far are concentrated in woody plants. Furthermore, large scale patterns are mostly based on published literature for meta‐analyses, but direct measurements of leaf nutrient resorption in environmental gradients are scarce. It remains imperative to employ natural gradients of climate and nutrient availability to elucidate the relationship between nutrient resorption and nutrient availability which is affected by biophysical factors (Brant & Chen, 2015). In addition, the functional types are separated according to growth forms, for example, as deciduous versus evergreen and tree shrubs versus graminoids. The difference of plant functional groups at species level is necessary to explore idiosyncratic plasticity of nutrient resorption in response to environment gradients.

Now there are few studies of leaf nutrient resorption in alpine meadows of east Tibet (Jiang et al., 2012; Liang, Zhang, & Zhang, 2015). Our team explored leaf nitrogen resorption efficiency of *Stipa purpurea* along the precipitation gradient (Zhao et al., 2016). The rich function groups of widespread herbs provide opportunities for studying the patterns of species‐specific nutrient resorption along environment gradients, for example, across precipitation amplitude. However, we still lack a predictive understanding of the main and interactive effects of soil nutrient availability, leaf nutrient stoichiometry on the nutrient resorption of different functional groups. The main objectives of this study were to: (1) assess leaf N and P resorption patterns along the precipitation gradient; (2) discriminate species‐specific difference among functional groups; and (3) determinate what are the main controls of these patterns. We hypothesized that the patterns of N and P resorption efficiencies would increase in N‐ and P‐poor environment, respectively, which would be regulated by precipitation and differentiated by functional groups along a precipitation gradient. To test these hypotheses, we chose four dominant and common species of functional groups as grass, sedge, forb, and legume to explore the patterns and controls of leaf nutrient resorption along a precipitation gradient on the Changtang Plateau, North Tibet. This study would provide valuable insights into the nutrient conservation strategies of dominant functional groups, which in turn may affect the nutrient cycling in this nutrient deficient environment.

## 2 MATERIALS AND METHODS

### 2.1 Study area

Changtang Plateau is the main part of Tibetan Plateau, locating in northwest Tibetan Autonomous Region, China (29°53′–36°32′N; 78°41′–92°16′E) with an average altitude of 4,400 m. A remarkable precipitation gradient (<100–700 mm) spans 1,500 km with successive grasslands of alpine desert, steppe and meadow from west to east. The alpine vegetation is dominated by alpine steppe with widespread species of *Stipa purpurea* Griseb., *Carex moorcroftii* Falc. ex Boott, and a variety of forbs (Li, 2000; Wu, Shen, & Zhang, 2014). N‐fixed legumes, for example, species of *Oxytropis* are common in western arid side of the Plateau. Soil nutrient is relatively low, with soil organic matter increase from <1.0% to 4.0% and total N (TN) from 0.02% to 0.2%, respectively (Li, 1980). Soil nutrient closely coupling with soil moisture in the precipitation gradient plays an important role in nutrient cycling in alpine grasslands. The Plateau is characterized by a cold, arid and windy climate, and sparse, vulnerable vegetation (Li et al., 2011). The general evaporation strength is larger than 1,800 mm, annual mean wind speed is more than 3 m/s, and annual mean aridity index is in the range of 1.6–20 (Mao, Lu, Zheng, & Zhang, 2006). It is cold on the Plateau with an annual mean temperature (AMT) of less than 0°C, and an annual temperature in the warmest month (July) of less than 14°C in most of the area (Yang, Zhang, Miao, & Wei, 2003). The longitudinal change of AMT is less than 2°C despite substantial precipitation range in 32°N latitude sampling transect in the Changtang Plateau.

### 2.2 Field sampling and laboratory analysis

The growing season usually begins in May and ends in September in the Plateau. Most herbaceous plants generally reach their peak coverage and growth in late July or early August, and senesce in September (Wu, Zhang, et al., 2014). Therefore, we carried out transect survey twice to sample green and senesced leaves at 12 sites ranging from alpine desert, steppe an meadow across the Plateau, respectively at the mid (late July) and the end (early October) of growing season in 2014 (Table 1). To cover precipitation and resources gradients as wide as possible, the distance between any two adjacent sites is controlled at least within 50–80 km. We chose four typical four species of the main functional types: legume (*Oxytropis* sp.), grass (*S. purpurea*), sedge (*C. moorcroftii*), and forb (*Potentilla bifurca* L), which were present at most sites with sufficient individuals for sampling.

**Table 1:** The location and environmental characteristics of sampling sites

Site	Latitude (°)	Longitude (°)	Elevation (m)	MAP (mm)	MAT (°C)	Common species
1	31.5882	91.6590	4,635	525.44	−0.4	*Stipa purpurea*,* Carex moorcroftii*,* Potentilla bifurca*
2	31.3971	90.8138	4,619	466.13	0.1	*S. purpurea*
3	31.3942	90.3135	4,632	432.63	0.2	*S. purpurea*,* C. moorcroftii*
4	31.6226	89.4819	4,660	394.95	−0.7	*S. purpurea*,* P. bifurca*
5	31.7149	88.5858	4,558	366.65	−1.0	*S. purpurea*
6	31.8696	87.8611	4,570	344.11	−1.4	*S. purpurea*
7	31.7940	87.3316	4,557	327.59	−0.9	*S. purpurea*,* C. moorcroftii*,* Oxytropis* sp.
8	32.0846	86.9078	4,615	310.80	−1.5	*S. purpurea*,* O. *sp., *P. bifurca*
9	31.9039	86.3425	4,756	291.65	−0.8	*S. purpurea*
10	31.9944	85.5666	4,928	261.1	−0.6	*S. purpurea*,* C. moorcroftii*,* O. *sp.
11	31.9949	84.8298	4,591	230.18	0.6	*S. purpurea*,* C. moorcroftii*,* O. *sp.*, P. bifurca*
12	32.2682	84.3156	4,498	204.25	0.7	*S. purpurea*,* O. *sp.

Species of aforementioned four functional groups were selected to collect leaf samples in each site (Table 1), from which at least 20 healthy plant individuals with mature and fully extended green leaves were randomly selected with five replicates at 500‐m intervals in late July, and the senesced leaves were sampled in the same way in early October. Furthermore, three soil profiles were randomly selected from each site at 500‐m intervals. Soil samples (0–20 cm depth) were collected from each soil profiles.

All leaf samples were oven‐dried at 65°C for 48 hr to constant weight and ground using a mill before passing through a 60‐mesh screen. The soil samples were ground to a 100‐mesh sieve after air‐drying. The C/N analyzer (Elementar Vario Max, Germany) was used to test leaf N concentration and soil total N, and the sulfuric acid–perchlorate acid heating digestion method was used to measure leaf P concentration and total soil P (TP).

### 2.3 Data processing and statistical analysis

Leaf nutrient resorption efficiency (RE) refers to percentage reduction in a nutrient between green leaves (LN_g_, g/kg) and senesced leaves (LN_s_, g/kg), calculated as (LN_g_–LN_s_)/ LN_g_ ×100%. Nutrient resorption proficiency (RP) is the nutrient concentration in senesced leaves (LN_s_) collected at the end of the growing season, which is considered the direct index for plant nutrient resorption capacity (Killingbeck, 1996). Nutrient resorption capacity is considered as higher efficiency when RE is higher and as higher proficiency when LN_s_ is lower.

The precipitation and temperature data were obtained from national meteorological observatories and HOBO automatic weather stations built in the Plateau by the Lhasa Plateau Ecological Experimental Station, Chinese Academy of Sciences.

Nested analyses of variances (ANOVAs) based on the decomposition of Type I sums of squares, were performed to partition variance components of nutrient resorption across different functional types and different sites (Nested Procedure, SAS version 9.1; SAS Institute Inc., Cary, NC, USA) (Liu et al., 2010). ANOVA was used for comparing the mean value differences of different functional types. Correlation between nutrient resorption efficiencies and environmental factors was analyzed using regression analyses. All statistical analyses were performed using SPSS version 17.0 software (SPSS Inc., Chicago, IL, USA).

Standardized major axis (SMA) regression was used to quantify relationships between N:P ratio of green leaves ([N:P]_g_) and leaf nutrient resorption across different functional types. All data were log transformed to satisfy the normal distribution before analysis. The DOS‐based SMATR package allows testing for homogeneity among SMA slopes via a permutation test (Falster, Warton, & Wright, 2006).

Considering substantial differences in N concentration and NRE between legumes and nonlegumes, and in P concentration and PRE between sedges and other species (Table 2), we separated legume and sedge, respectively from the remaining species in controlling factor analysis for N and P resorption (see Results for detail). Structural equation modeling (SEM) is a multivariate statistical technique to analyze structural relationships between measured variables and latent constructs. SEM combines factor analysis and multiple regression analysis and estimates the multiple and interrelated dependence in a single analysis (Grace & Pugesek, 1997; Shipley, 2001). To evaluate the contributions of different factors to leaf nutrient resorption, we conducted a theoretical structure relating direct and indirect relationships among these factors and leaf nutrient resorption, and tested significance using SEM. The pathway analysis was performed with Amos 17.0 program (SPSS Inc., Chicago, IL, USA). The maximum likelihood estimate was used to calculate the standard path coefficients between different variables, which produced standard regression coefficients and the probabilities. The chi‐square test was used to verify the fitness of the statistical modeling. A insignificant goodness of fit chi‐square test indicates that the model fits the data. If a model was not rejected and considered as biologically and ecologically plausible, parameter estimates can be used to study direct and indirect effects (Vile, Shipley, & Garnier, 2006).

**Table 2:** Mean N and P concentrations and N:P ratios of green and senesced leaves for different plant functional groups

Functional group	N_g_ (g/kg)	N_s_ (g/kg)	P_g_ (g/kg)	P_s_ (g/kg)	[N:P]_g_	[N:P]_s_	NRE (%)	PRE (%)
Grass (*S. purpurea*)	24.28 ± 1.26^a^	6.17 ± 0.30^a^	0.82 ± 0.10^a^	0.17 ± 0.02^a^	44.05 ± 12.34^a^	40.1 ± 3.17^a,b^	74.4 ± 0.7^a^	72.6 ± 6.0^a,b^
Sedge (*C. moorcroftii*)	25.90 ± 1.92^a^	6.8 ± 0.59^a^	1.08 ± 0.08^a^	0.14 ± 0.01^a^	24.36 ± 2.28^a^	49.8 ± 3.58^a^	73.7 ± 1.5^a^	87.4 ± 0.5^a^
Forb (*P. bifurca*)	28.06 ± 1.79^a^	7.38 ± 0.26^a^	1.77 ± 0.04^b^	0.47 ± 0.15^b^	15.86 ± 1.09^a^	21.72 ± 6.55^b^	73.5 ± 1.0^a^	73.3 ± 8.5^a,b^
Legume (*O. *sp.)	33.93 ± 1.91^b^	18.00 ± 1.58^b^	1.28 ± 0.21^a^	0.42 ± 0.05^b^	31.99 ± 8.49^a^	46.4 ± 8.27^a^	47.2 ± 1.9^b^	62.0 ± 9.0^b^

Values are presented as mean concentrations ± standard error.

Within any column, different letters indicate significant differences (*p *<* *.05) between functional types based on post hoc comparisons (Turkey HSD tests).

## 3 RESULTS

### 3.1 The variations of foliar N, P, and RE across sites and functional types

**Figure 1:**
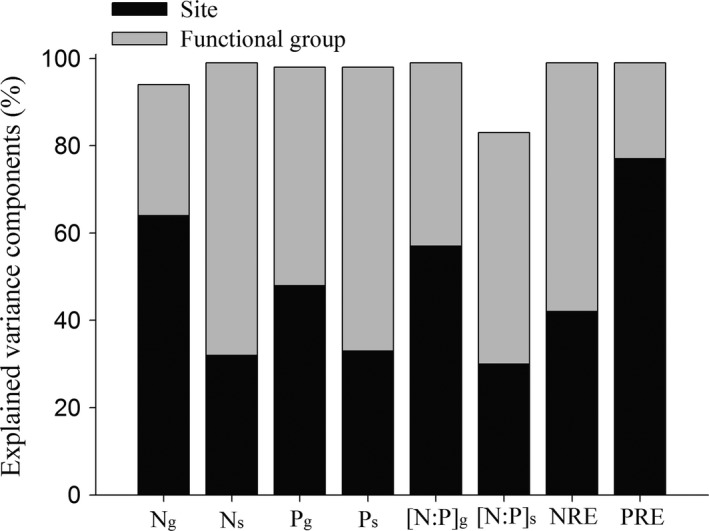
Contribution of different sites and plant functional groups to variance of leaf N, P, and resorption efficiency. Organism photograph: A typical alpine steppe dominated by widespread species of *Stipa purpurea* Griseb and Tibetan gazelle

Remarkable differences were found in leaf nutrient concentration and resorption efficiency across functional types and sites along the precipitation gradient on the Plateau (*p *<* *.01). The contribution of functional types and sites to variances changed with the indices considered (Figure 1). The variances of the N concentration in green leaves (N_g_), [N:P]_g_, and PRE were explained more by sites than by functional types, whereas variances of the remaining indices were more explained by functional types (Figure 1).

Nitrogen‐fixed legumes had significantly higher mean N_g_, N_s_, and lower mean NRE than nonlegumes. There were no differences among nonlegumes (Table 2). Grass and sedge had significantly lower mean P concentrations in green leaves (P_g_) and senesced leaves (P_s_) than legume and forb. The mean PRE was highest in sedge but lowest in legumes (Table 2). No differences in mean [N:P]_g_ were found among functional types. However, forbs had a remarkably higher [N:P]_s_ than the other species (Table 2).

### 3.2 The patterns of soil, leaf nutrient, and leaf nutrient resorption along the precipitation gradient

**Figure 2:**
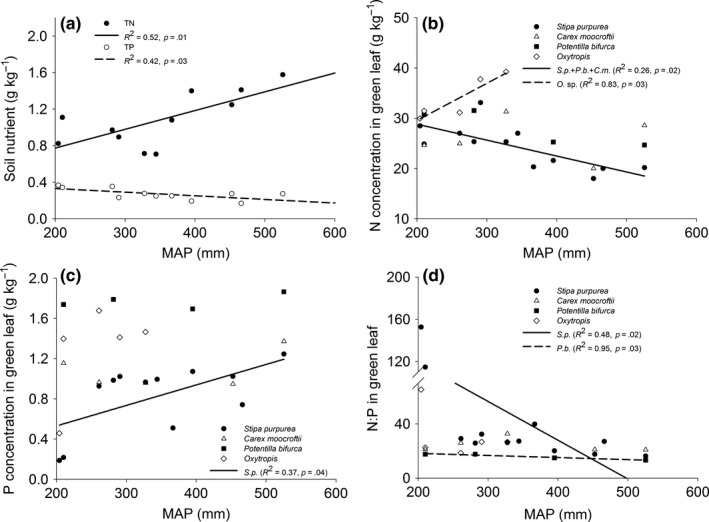
Changes of N and P concentration of soil and green leaves along the precipitation gradient

Soil TN (with a mean of 1.23 g/kg) increased, while TP (with a mean of 0.27 g/kg) decreased with increasing MAP (Figure 2a). The N_g_ of nonlegumes decreased but N_g_ of legumes (*Oxytropis* sp.) increased with increasing MAP (Figure 2b). Only the P_g_ of *S. purpurea* increased with MAP (Figure 2c). The [N:P]_g_ of *S. purpurea* and *P. bifurca* decreased with MAP, but the latter decreased very gently (Figure 2d).

**Figure 3:**
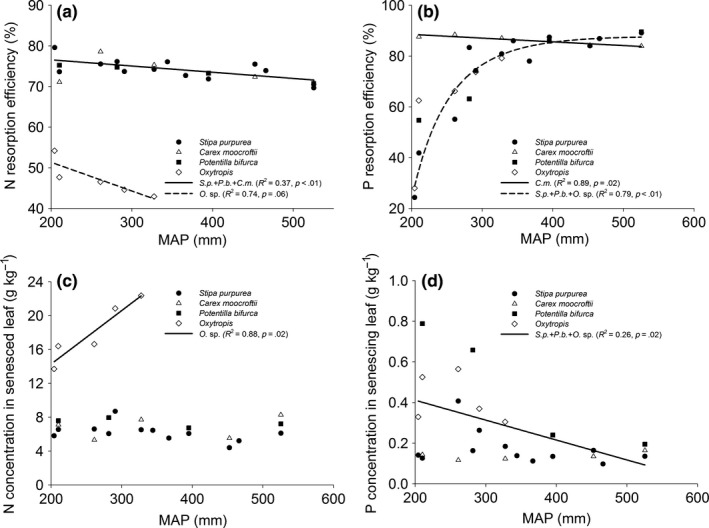
Variation of leaf nutrient resorption efficiency and nutrient resorption proficiency along the precipitation gradient

N resorption efficiency decreased with increasing MAP. But *Oxytropis* sp. had much lower and sharper decline NRE than other species (Figure 3a). The PRE of sedge, *C. moorcroftii* decreased linearly while those of other species increased asymptotically with increasing MAP (Figure 3b). The N_s_, namely NRP, of legume was relatively high and increased with increasing MAP in arid side, whereas those of nonlegumes had lower values and exhibited no trends in precipitation gradient (Figure 3c). Except for sedge, the remaining species had decreasing P_s_, that is, PRP with MAP (Figure 3d).

### 3.3 The influence of soil and leaf nutrient on leaf nutrient resorption

**Figure 4:**
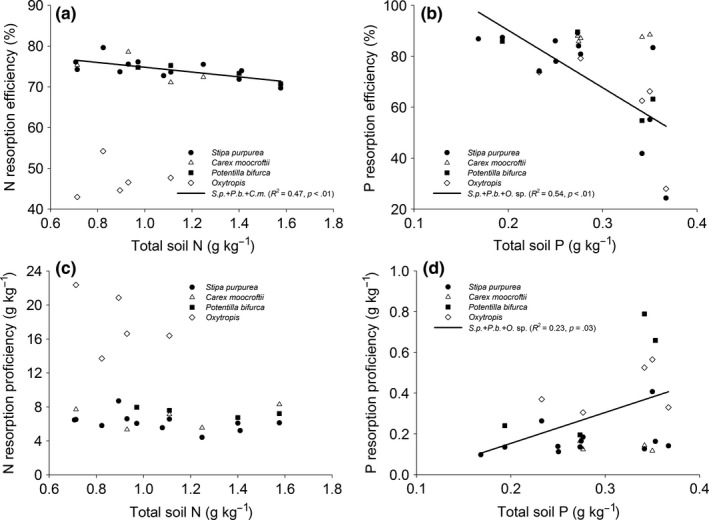
Relationship between nutrient resorption efficiency, nutrient resorption proficiency, and soil nutrient content

**Figure 5:**
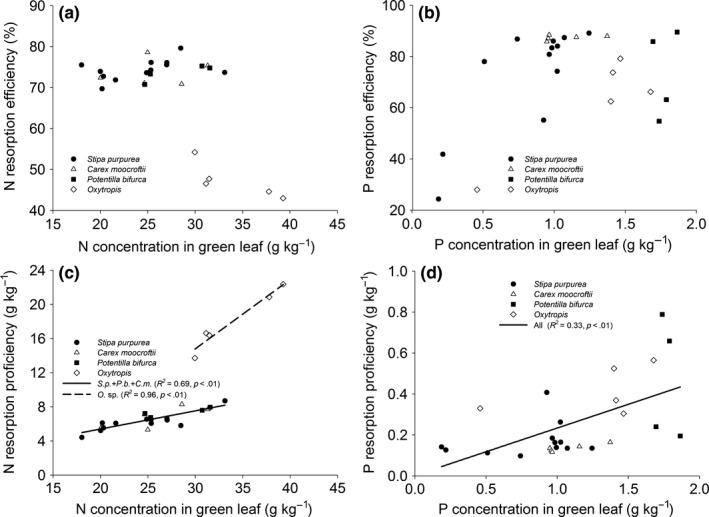
Relationship between nutrient resorption efficiency, nutrient resorption proficiency, and leaf nutrient concentration

Except for legume, NRE, but not NPR was negatively correlated with TN. Compared with nonlegumes, legume had lower levels of NRE and NRP under the same TN (Figure 4a, c). Except for sedge, that is, *C. moorcroftii*, the PRE and PRP of other species decreased and increased, respectively with TP. (Figure 4b, d). There was no correlation between NRE and N_g_ (Figure 5a). However, legume exhibited sharper increase in NRP with N_g_ than other species (Figure 5c). PRP but not PRE increased with P_g_ (Figure 5b, d).

### 3.4 The influence of leaf N and P stoichiometry on leaf nutrient resorption

Standardized major axis regression showed that NRE significantly increased and PRE (excluding sedge) decreased with [N:P]_g_, respectively. Significant difference existed among the slopes of RE against [N:P]_g_ across functional types, indicating species‐specific response to [N:P]_g_. However, NRP was not strongly correlated with [N:P]_g_ in nonlegumes and thus no prediction could be made for the changes in NRP with [N:P]_g_. PRP was correlated with [N:P]_g_ except for *S. purpurea* (Table 3).

**Table 3:** Summary of standardized major axis regression parameters relating N and P resorption efficiencies and proficiencies to N:P ratios in green leaves

	Functional group	*n*	*R* ^2^	*p*	Slope	Slopes homogeneity (*P*)
NRE (%) vs. Log [N:P]_g_	*Stipa purpurea*	36	0.20	.03	0.10	<0.01
	*Carex moorcroftii*	15	0.52	.02	0.33	
	*Potentilla bifurca*	12	0.53	.04	0.27	
	*Oxytropis* sp.	15	0.55	.01	0.21	
PRE (%) vs. Log [N:P]_g_	*S. purpurea*	36	0.81	<.01	−0.70	<0.01
	*C. moorcroftii*	15	0.35	.07	0.23	
	*P. bifurca*	12	0.61	.02	−2.34	
	*O*. sp.	15	0.65	<.01	−0.94	
Log Ns vs. Log [N:P]_g_	*S. purpurea*	36	0.02	.53	0.25	<0.01
	*C. moorcroftii*	15	0.01	.80	0.85	
	*P. bifurca*	12	0.52	.05	0.53	
	*O*. sp.	15	0.23	.16	−0.41	
Log Ps vs. Log [N:P]_g_	*S. purpurea*	36	0.02	.53	−0.58	<0.01
	*C. moorcroftii*	15	0.58	.01	−1.26	
	*P. bifurca*	12	0.56	.03	4.21	
	*O*. sp.	15	0.41	.05	−0.58	

### 3.5 The contribution of different controlling factors to leaf nutrient resorption

**Figure 6:**
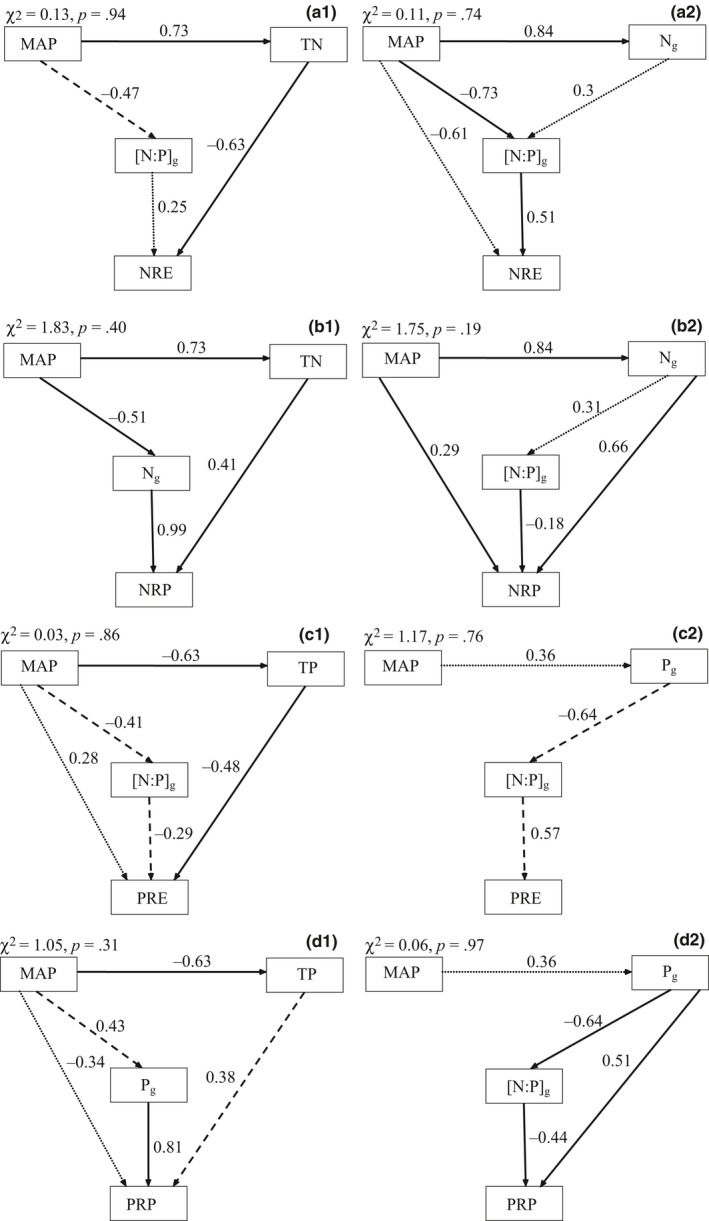
Controlling factor analysis of leaf resorption efficiency and proficiency using the structural equation model. Significant regressions are indicated by solid lines (*p *<* *.01), marginally significant by dashed lines (*p *<* *.05) and nonsignificant regressions by dotted lines. (a1 nonlegumes, a2 legume only; b1 nonlegumes, b2 legume only; c1 excluding *Carex moorcroftii*, c2 *Carex moorcroftii* only; d1 excluding *Carex moorcroftii*, d2 *Carex moorcroftii* only)

Structural equation modeling analysis showed that the optimal model of NRE for nonlegumes contained three factors: MAP, TN, and [N:P]_g_. NRE was determined largely by TN and marginally by [N:P]_g_. MAP had an indirect impact on NRE through influencing TN and [N:P]_g_ (Figure 6 a1). NRE of legume was mainly affected by [N:P]_g_ and MAP. MAP played an indirect role on NRE by influencing [N:P]_g_ (Figure 6 a2). The PRE of species except for *C. moorcroftii* was mainly impacted by TP. [N:P]_g_ had marginal impact on PRE and MAP indirectly regulated PRE through TP and [N:P]_g_ (Figure 6 c1). However, *C. moorcroftii* was mainly determined by [N:P]_g_. P_g_ brought about indirect impacts on PRE through influencing [N:P]_g_ (Figure 6 c2).

N_g_ was found to be essential for NRP, and TN had a remarkable impact on NRP with indirect regulation by MAP (Figure 6 b1). The NRP of the legume was also mainly influenced by N_g_, descendingly by MAP and [N:P]_g_ in sequence (Figure 6 b2). Similarly, in the optimal model of PRP (excluding *C. moorcroftii*), P_g_ had the most remarkable impact on PRP, while TP had marginal effect on PRP, and MAP had an indirect impact on PRP through TP and P_g_ (Figure 6 d1). While PRP of *C. moorcroftii* was mainly impacted by P_g_ and [N:P]_g_ (Figure 6 d2).

## 4 DISCUSSION

We found leaf N and P resorption patterns along the precipitation gradient in the Changtang Plateau, which supported our hypotheses that NRE decreased but PRE increased with increasing precipitation from west to east. The trends were also exhibited in different functional groups. The observed patterns of leaf nutrient conservation strategies were affected by soil nutrient conditions, leaf stoichiometry, and differentiated from functional species, which were regulated by precipitation.

### 4.1 N and P resorption patterns along the precipitation gradient

Along the precipitation gradient on the Plateau, soil TN increased while TP decreased with precipitation, indicating that precipitation plays a pivotal role in affecting soil nutrient availability (Drury et al., 2003; Paul et al., 2003; Wu et al., 2013). N is mainly derived from biogeochemical processes, and P is mainly derived from physical processes. At the more arid end of the precipitation gradient, N limitation is stronger whereas P limitation is weaker, and vice versa at the other wetter end (Wardle, 2013). This trend is in line with the previous reports in arid areas (Delgado‐Baquerizo et al., 2013; Emadi, Baghernejad, Bahmaniarand, & Morovvat, 2012; Wardle, 2013). In the soil nutrient gradient, the patterns of NRE decreased, but PRE decreased with increasing precipitation, suggesting that plants under N and P limitation are likely to increase N and P resorption from the low soil nutrient conditions in the Plateau. That is to say, plants in the nutrient‐limited environment adopt nutrient conservation mechanisms to minimize nutrient loss through internal nutrient cycling. This result is consistent with global‐scale patterns of N and P resorption associated with precipitation (Yuan & Chen, 2009), but contrary to the result of both PRE and NRE decreased with precipitation by Vergutz et al. (2012). This result suggests that N and P resorption is more divergently dependent on element limitation itself rather than soil fertility.

Our measured mean N_g_ of each functional type was higher than that reported in humid alpine meadow of east Tibet (Jiang et al., 2012; Liang et al., 2015) and the global average (Reich, Oleksyn, & Tilman, 2004; Vergutz et al., 2012), while mean P_g_ of each functional type was much lower than the global average (Reich et al., 2004). This result indicated alpine plants were more limited by phosphorus than nitrogen, and were likely to better adapt to alpine and infertile environment. Except for legume, both N and P resorption efficiencies of other functional types showed obviously higher values than the global‐scale average compiled by Aerts (1996) and Yuan and Chen (2009), suggesting very efficient nutrient conservation of alpine plants on the Plateau. Leaf nutrient resorption is considered highly proficient if N_s_ and P_s_ are below 7 and 0.5 g/kg, respectively, and as ultimate potential resorption if N_s_ and P_s_ as low as 3 and 0.1 g/kg, respectively (Killingbeck, 1996). Accordingly, alpine species had very high resorption of N and P, and sedge and grass showed almost ultimate resorption. The higher resorption efficiency and proficiency suggested that alpine plants were likely well adapted to nutrient‐limited environment through high internal N and P recycling (Freschet, Cornelissen, van Logtestijn, & Aerts, 2010; Norris & Reich, 2009). Moreover, the PRE of different functional types was higher than the NRE, indicated that P is more limited than N on the Changtang Plateau.

In this study, we took insufficient account of the impacts of leaf shrinkage and mass loss during senescence on resorption estimation. It is reported that leaf size change and mass loss may lead to considerable underestimation of resorption. Leaf shrinkage could result in an average underestimation of 6% when using area‐based concentrations (Van Heerwaarden, Toet, & Aerts, 2003). This suggests that the alpine plants in this study could have even higher resorption efficiencies than our measured. However, the size of the leaves during abscission may not change much in the arid and semiarid climate. We found that the amount of carbon loss in senesced leaves was small with an average less than 0.2%. Furthermore, our using the unit mass of nutrient concentration rather than the unit area of nutrient concentration is also effective to reduce the impact of leaf area changes. Therefore, this study may not lead to too much underestimation. Even though underestimation, our uncorrected resorption efficiencies for N and P were higher than the corrected world means of N (62.1%) and P (64.9%), respectively (Vergutz et al., 2012). Therefore, our study unraveled the fact of nutrient resorption higher on the Plateau than the world average. The only unbiased method estimate resorption is based on measurement of nutrient pools in the same leaves before and after senescence (Vergutz et al., 2012). However, due to the long‐distance sampling in different seasons, this expectation is difficult to be realized for nondestructive sampling. Nevertheless, leaf shrinkage and mass loss deserve to be considered in order to avoid of real estimation of resorption efficiency.

### 4.2 N and P resorption difference among functional groups

Nutrient resorption differed significantly among plant functional types on the Changtang Plateau. The legume had much higher mean N_g_, N_s_ but lower NRE than nonlegume species. Grass, sedge, and forb had much lower foliar N concentrations and higher leaf N resorption efficiencies. Interestingly, sedge had higher P resorption than the other functional groups. Our findings suggest great difference among functional group in nutrient resorption in the axis of resources along the precipitation gradient.

Legume is mainly distributed in the west arid end with MAP less than 350 mm. In the N‐limiting soil environment, legumes had a wealth of N‐fixing bacteria to fix N from atmosphere and thus highest N_g_. Therefore, nitrogen‐fixing legume reduces the demand for soil N (Liang et al., 2015; Yuan, Li, Han, Huang, Jiang, Wan, Zhang, et al., 2005), less affected by soil N availability, and adopted a progressive strategy. However, the other nonlegumes adopt more conservative N use strategy (Aerts & Chapin, 2000; Yuan, Li, Han, Huang, & Wan, 2005) by their internal N cycling to adapt to low nutrient environment (Freschet et al., 2010). Moreover, NRE of legume decreased more rapidly than nonlegumes (Figure 3a). This can be explained by N_g_ increase sharper than N_s_ in legume, but N_g_ decrease faster than N_s_ in nonlegumes. The reason why the N_g_ of legume increased with increasing precipitation is that the quantity and activity of N‐fixing bacteria increased with the increase in precipitation, resulting in an increasingly strengthened N‐fixation capacity (Aranibar et al., 2004).

The P_g_ of grass species increased with increasing MAP, whereas those of the other species were not strongly correlated with MAP (Figure 2c), indicating the limited change of P_g_ for most species under P‐limited environment. However, P_s_ increased with soil P except for sedge (Figure 4d). As a result, grass, forb, and legume had decreasing PRE in response to increasing soil P. But in terms of their response to precipitation, a stronger increase in PRE with precipitation was exhibited before a threshold of 400 mm. This result indicates alpine deserts and steppes are less P limited than alpine meadows, which can be explained by higher P availability in more arid areas due to stronger physical weathering while greater P limitation in more humid areas due to biochemical limitation (Delgado‐Baquerizo et al., 2013). Previous studies in Tibetan Plateau also supported our results (Hong, Wang, & Wu, 2015; Jiang et al., 2012; Liang et al., 2015). The result also suggests grass, forb, and legume exhibited high plasticity in response to soil phosphorus change in arid and semiarid environment.

In contrast, sedge had very high PRE but showed a slight decrease with MAP (Figure 3b). This implies that on the one hand, sedge is most efficient in phosphorus conservation in P‐limited environment on the Plateau, and on the other hand, sedge might shift N resorption in the more aid end to P uptake in more humid end. Although our few data points showed no significant trends of P_g_ and P_s_ to support above speculation, it was directly supported by the evidence that sedge had deep and looser roots in arid side but shallow and denser roots mass in humid side on the Plateau (data not shown) and previous study (Fort, Jouany, & Cruz, 2013). The significant higher PRE of sedge in the precipitation gradient was due to very low P_s_ rather than higher P_g_, which is considered trade‐off between them (Deng et al., 2016). Liang et al. (2015) also show that graminoids have the lowest nutrient in senesced leaves. Moreover, the sedge leaf habit of creeping rhizomes and dense roots might dilute the phosphorous contents in leaves in P‐limited environment. Further, sedges have low P uptake in the low soil P availability (Perez‐Corona & Verhoeven, 1996). And highly dense root systems in end of season might be great sink of P. This is the reason why P_s_ was lowest in sedge compared with in other functional groups. Overall, all these reasons result in high P resorption of sedge on the Plateau. Therefore, the sedge species have a greater competitive advantage in nutrient‐poor environments than other functional groups (Gusewell, 2004). This further explains why the sedges are the dominant functional groups in alpine regions.

### 4.3 Controls of N and P resorption

#### 4.3.1 Influence of climate and soil conditions

Although MAP influenced leaf nutrient resorption in the precipitation gradient, results of SEM showed that soil N and P directly affected and contributed more explanation to N and P resorption of most species. This result suggests soil nutrient exerts a dominant control of NRE and PRE, while precipitation indirectly influences resorption through soil nutrient status, which is consistent with most of previous studies (Aerts, 1996; Aerts & Chapin, 2000; Yuan & Chen, 2015). Soil nutrient is essential for determining nutrient resorption in grasslands (Aerts & Chapin, 2000). Plants grown in nutrient‐poor environments have higher resorption capacity than those in nutrient‐rich environments (Killingbeck, 1996; Ralhan & Singh, 1987; Wright & Westoby, 2003). Plants either absorb soil nutrients or resorb nutrients from their own senesced tissues, with processes including a series of cost input mechanisms (Aerts & Chapin, 2000; Rejmánková, 2005). Nutrient resorption capacity is actually a trade‐off between the costs of soil nutrient uptake and leaf nutrient resorption (Ratnam et al., 2008). With the increase in soil nutrients content, the cost of nutrient resorption from senesced tissues is increasing, while the relative cost of directly soil nutrient uptake is decreasing (Wright & Westoby, 2003).

#### 4.3.2 Influence of leaf nutrient

Resorption efficiency was not, while RP was positively correlated with leaf nutrient along the precipitation gradient, indicating that leaf nutrient had no remarkable impact on RE. This result is in accordance with the conclusion of Aerts (1996). It is generally assumed that species in nutrient‐poor environment have low leaf nutrient concentrations, low tissue turnover rates, and high nutrient resorption efficiencies (Aerts & Chapin, 2000). The lower nutrient in green leaves, the lower the nutrient would be in senesced leaves, and thus higher nutrient resorption in drier and poor nutrient environment (Kobe et al., 2005; Ratnam et al., 2008). On the contrary, plants had higher N concentrations in green leaves in the arid areas on the Plateau. This is partly because drought stress strengthens the protection of internal water contents by increasing N input to the nonphotosynthetic tissues in leaves and increases osmotic pressure in cells in order to better adapt to arid environment (Osmond et al., 1987; Seligman & Sinclair, 1995). As a consequence, a higher N_g_ occurred in the more arid and infertile environment on the Plateau (Zhao et al., 2016). In fact, the impact of leaf nutrient on nutrient resorption depends not only on leaf nutrient concentration but also on the proportion of soluble and insoluble nutrients in the leaves (Lajtha, 1987; Pugnaire & Chapin, 1993). The higher the concentration of soluble nutrients in green leaves, the higher the RE would be (Pugnaire & Chapin, 1993). However, many species in nutrient‐poor environment also have a higher soluble nutrients concentration (Côté, Vogel, & Dawson, 1989; Navari‐Izzo, Quartacci, & Izzo, 1990). Therefore, leaf nutrient cannot be predicted by nutrient limitation in arid or semiarid ecosystems, and leaf resorption is not necessarily correlated with leaf nutrient (Newman & Hart, 2015).

#### 4.3.3 Influence of leaf nutrient stoichiometry

Previous studies have shown that the higher the [N:P]_g_, the higher the P limitation would be in contrast with N limitation, and vice versa (Gusewell, 2004; Tessier & Raynal, 2003). Therefore, with increasing [N:P]_g_, P resorption capacity should increase, whereas N resorption capacity should decrease. However, this study on the contrary showed that NRE increased while PRE decreased with increasing [N:P]_g_. Along the precipitation gradient on the Changtang Plateau, TN increased while TP decreased with increasing precipitation (Figure 2b), in line with the previous studies of increasing soil N availability (Drury et al., 2003; Paul et al., 2003; Wu et al., 2013) and decreasing soil P availability with increasing precipitation (Delgado‐Baquerizo et al., 2013; Wardle, 2013). In general, plant tissues with higher soil nutrient availability are considered to have higher nutrient content (Kobe et al., 2005; Yuan, Li, Han, Huang, Jiang, Wan, et al., 2005). However, the extremely arid climate resulted in more N input to the leaves at dry end on the Changtang Plateau (Figure 2b) (Mao et al., 2006; Osmond et al., 1987; Seligman & Sinclair, 1995), while the leaf P concentration of different functional types decreased or did not change significantly with decreasing precipitation (Figure 2c). In nutrient‐poor environments, plant leaves should have a higher NRE at the dry end and a higher PRE at the humid end to maintain a consistent or higher input of N and P. The change in [N:P]_g_ through the precipitation gradient showed a greater difference of N and P inputs in plant leaves than the relative limitation strengths of N and P. The changing [N:P]_g_ in the precipitation gradient on the Changtang Plateau shows more of a difference in N and P input to plant leaves than the relative limitation strengths of N and P. Therefore, it is unreasonable to deduce the relative limiting strengths of N and P by [N:P]_g_ changes in the arid or semiarid ecosystem (Drenovsky & Richards, 2004; Ratnam et al., 2008; Rejmánková, 2005).

## 5 CONCLUSION

Our study indicated a decrease N resorption but an increase P resorption with increasing precipitation on the Changtang Plateau, Tibet. P was proved to be more limited than N for plant nutrient use and growth especially in the eastern humid end. Both N and P resorption exhibited higher levels compared with the world average, indicating very proficient nutrient conservation of alpine grassland plants. Distinct differences of nutrient resorption exist among plant function groups. Specifically, legumes had higher leaf N concentration but the lowest resorption efficiency, while sedge had the highest P resorption efficiency. Leaf nutrient resorption efficiencies of N and P were largely controlled by soil nutrient availability, leaf stoichiometry and indirectly regulated by precipitation. The different patterns of species‐specific N and P resorption have important impact on not only nutrient conservation but also species composition and distribution.

## CONFLICT OF INTEREST

None declared.
